# A Latent Profile Analysis of Emotions in AI-Mediated IDLE: Associations with Emotion Regulation Strategies and Perceived AI Affordances

**DOI:** 10.3390/bs16020283

**Published:** 2026-02-15

**Authors:** Zihan Gao, Chenxi Du

**Affiliations:** 1 School of Languages and Communication Studies, Beijing Jiaotong University, Beijing 100044, China; zhgao@bjtu.edu.cn; 2 School of Marxism, Beijing Jiaotong University, Beijing 100044, China; 3 School of International Studies, University of International Business and Economics, Beijing 100029, China

**Keywords:** AI-mediated IDLE, achievement emotions, cognitive reappraisal, expressive suppression, perceived AI affordances, latent profile analysis

## Abstract

The rapid development and easy accessibility of artificial intelligence (AI) technology have led to a significant rise in informal digital learning of English (IDLE). However, the emotional experiences across different cohorts of learners remain underexplored. Contextualized in AI-mediated IDLE, the present study integrated the control-value theory of achievement emotions and the process model of emotion regulation to investigate the latent profiles of emotions and further examine their relations to emotion regulation strategies (cognitive reappraisal and expressive suppression) and perceived AI affordances. Questionnaires were administered to 613 English as a foreign language undergraduates in China. Latent profile analysis revealed three emotion profiles, including moderate positive and moderate negative emotions group (Profile 1, 43%); high positive and low negative emotions group (Profile 2, 21%); and high positive and high negative emotions group (Profile 3, 36%). The Bolck–Croon–Hagenaars (BCH) analysis indicated that students in Profile 2 scored the highest on perceived AI affordances, followed by those in Profile 3 and Profile 1. Additionally, multinomial logistic regression analysis showed that cognitive reappraisal was a stronger predictor of membership in Profiles 2 and 3 compared with Profile 1, while expressive suppression predicted membership in Profile 3 to the greatest extent, followed by Profiles 1 and 2. Pedagogical implications were provided to cultivate learners’ optimal emotional state.

## 1. Introduction

The “affective turn” (Prior, 2019, p. 516) spurred by positive psychology has motivated scholars to explore both the positive and negative emotions in English as a foreign language (EFL) learning during the past decade (H. Liu et al., 2025a; Shao et al., 2020; Teng & Pan, 2024). Rather than attending to the overall trends in emotional variables across the whole sample, emotion profiles that identify subgroups of participants sharing similar emotion combinations have attracted increasing scholarly attention (Zhu et al., 2024). The control-value theory (CVT) of achievement emotions (Pekrun, 2006) posits the coexistence of positive and negative emotions. Existing studies have also revealed distinct emotion profiles (e.g., a positive emotions-driven profile with higher positive and lower negative emotions, a negative emotions-driven profile with higher negative and lower positive emotions, and a bimodal emotions-driven profile with both high positive and negative emotions) among EFL learners and demonstrated their significant associations with motivation (Tsang & Yeung, 2024) and English achievement (Shi & Wang, 2025).

Given that emotion profiles are context-specific (Pekrun et al., 2011), it is necessary to examine emotion profiles not only in face-to-face (F2F) classrooms (Tsang & Yeung, 2024; Y. Wang & Xu, 2024) and pre-exam contexts (Shi & Wang, 2025) but also in emerging technology-enhanced settings, such as AI-mediated informal digital learning of English (IDLE). IDLE is crucial because of EFL learners’ widespread adoption of digital tools (e.g., AI tools) outside class (G. Liu et al., 2025a; C. Wang et al., 2025). In addition, the novel features of these tools render emotions distinct from those in the F2F context (Lee & Chiu, 2023). An emphasis on AI-mediated IDLE also echoes the call to integrate emerging technologies in language education and nurture students’ autonomous and personalized learning (Soyoof et al., 2023). Despite its importance, research on emotions in AI-mediated IDLE remains dominated by variable-centered approaches, focusing on their antecedents, such as ideal L2 self and ought-to L2 self (G. Liu et al., 2024) and AI literacy (Zou et al., 2026), as well as their effects on various learning-related outcomes, such as IDLE engagement (G. Liu et al., 2025a; Zou et al., 2025b) and willingness to communicate (Zou et al., 2026). Additionally, emotions in these studies were largely confined to enjoyment (G. Liu et al., 2025c; Zou et al., 2025b). As a result, little is known about the co-occurrence patterns of multiple emotions across learner subgroups in AI-mediated IDLE contexts, leaving the person-centered, context-sensitive emotional landscape largely underexplored.

In addition to investigating the context-specific emotion profiles, it is crucial to identify the optimal emotion profile by examining consequences, one of which is perceived AI affordances. Perceived AI affordances refer to learners’ perception of the possibilities that AI tools can provide via human–AI interaction to support effective learning (Huang et al., 2025; Xu & Li, 2024). Empirical research has revealed that perceived AI affordances significantly influence EFL learners’ satisfaction of AI tools, increase their intention to use them and continuous usage (Huang & Zou, 2024; G. Liu et al., 2025a), and fundamentally shape their cognitive and behavioral engagement, which serves as a catalyst for enhancing learning motivation and enjoyment (G. Liu et al., 2025c) and increasing learning performance (G. Liu et al., 2025a). Therefore, investigating perceived AI affordances is warranted, as it provides a basis for understanding the prerequisites for human–AI collaborative learning behavior in the digital age. Previous studies have mainly examined the association between enjoyment and perceived AI affordances in EFL learning with a variable-centered approach (Huang & Zou, 2024; C. Wang et al., 2025). However, how emotion profiles impact perceived AI affordances remains unexamined, which constrains the understanding of which emotion combination places learners in an optimal emotional state for effective learner–AI collaboration.

To achieve facilitative emotion combinations, it is necessary to further illuminate the proximal antecedents (i.e., emotion regulation strategies, ERSs) for targeted interventions. The process model of emotion regulation (PMER) (Gross & John, 2003) assumes that emotions can be influenced by two crucial ERSs of cognitive reappraisal (reinterpreting a situation that could potentially trigger emotions) and expressive suppression (inhibiting ongoing emotional expressions), with contrasting influencing mechanisms. Despite the evidence for the association between ERSs and separate emotions (Alqarni, 2024; Jiang et al., 2025), the effects of the two strategies on complex emotion co-occurrence patterns remain unexplored.

To address the aforementioned gaps, the present study aims to explore the profiles of diverse emotions, including positive emotions (enjoyment and hope) and negative emotions (anxiety and disappointment) in AI-mediated IDLE, as well as their associations with ERSs (cognitive reappraisal and expressive suppression) and perceived AI affordances. The present study, therefore, advances existing research in three key respects. First, it integrates the CVT and the PMER to examine the ERSs–emotions–emotional outcomes nexus, offering a more comprehensive theoretical account of how emotions emerge and function in EFL learning. Second, moving beyond the predominant variable-centered approach, it adopts a person-centered approach to capture the co-occurrence patterns of multiple emotions. Third, it extends the emotion profile research into the emerging AI-mediated IDLE context, thereby enriching the understanding of learners’ emotional experiences and offering insights for improving learner–AI collaborative effectiveness. Pedagogically, it enables EFL instructors to understand students’ distinct emotion combinations when adopting AI tools to learn English outside class and provide targeted guidance on emotion regulation. In this way, an optimal emotional state can be achieved to facilitate learners’ perceptions of AI affordances and ultimately enhance learning performance.

## 2. Literature Review

### 2.1. Theoretical Framework

The present study integrated the CVT (Pekrun, 2006) and the PMER (Gross, 2015) to investigate the emotion profiles, as well as their antecedents (the ERSs of cognitive reappraisal and expressive suppression) and outcomes (perceived AI affordances) in AI-mediated IDLE. Specifically, the study draws upon the CVT to hypothesize the different emotion profiles. Proposed by Pekrun (2006), the CVT is the most widely applied framework for emotion research, which has been adopted in the Chinese EFL learning context (Li et al., 2020), as well as the IDLE context in China (Zou et al., 2025a), indicating its applicability across sociocultural and technological contexts. The CVT particularly assumes the “simultaneous or alternating experiences” of emotions (Pekrun, 2006, p. 321), supporting the claim in the present study that learners may experience combinations of emotions. In addition, the types and intensity of emotions and, thereby, the proportion of emotion combinations are shaped by contextual factors, one of which is the sociocultural context (Pekrun, 2006). For example, Chinese EFL learners in Dewaele and Li (2022) reported higher levels of anxiety than the international samples in Dewaele and MacIntyre (2014), partly due to the fact that the exam-oriented, performance-driven nature of English learning in China decreased learners’ perceived control over and the intrinsic value of learning activities and intensified their anxiety. In addition, the learning medium itself shapes emotional experiences. Digital tools, as distal environmental factors, affect learners’ control-value appraisals, which in turn influence their emotions (Davis, 1989; Pekrun, 2006), particularly in the case of novel AI tools that introduce both new opportunities (e.g., instant communication and personalized feedback) and challenges (e.g., biased responses and a lack of social presence) (G. Liu et al., 2025a; Yang & Zhao, 2024). Therefore, due to the coexistence of different emotions and the context-specific nature of emotions assumed in the CVT, this study hypothesizes the presence of distinct emotion profiles in the AI-mediated setting among Chinese EFL learners.

The CVT (Pekrun, 2006) is also adopted to hypothesize the effect of emotion profiles on perceived AI affordances, conceptualized in four dimensions of interactivity (instant response and user-friendly interface), personalization (customization to individual needs), convenience (accessible and efficient learning experience), and social presence (human-like and emotionally supportive feedback) (Xu & Li, 2024). Particularly, it assumes that emotions influence learners’ cognitive resources and self-regulation of learning (Pekrun, 2006). According to Pekrun (2006), “general functional mechanisms of human emotions are bound to universal, species-specific characteristics of our mind” (p. 329), suggesting that the mechanism through which emotions influence learning processes can be applied to AI-mediated IDLE in China. In this regard, positive emotions expand cognitive flexibility and attention scope and enhance task-relevant thinking (Pekrun et al., 2023), enabling learners to channel sufficient cognitive resources to engage in learner–AI collaborative practices, making it easy for them to perceive AI’s interactivity affordances and convenient usage. In addition, positive emotions promote self-regulation of learning, which is particularly salient in IDLE, where learning is highly autonomous, learner-driven, and temporally and spatially flexible (Lee, 2019). With enhanced self-regulation, learners engage in goal-oriented learning behavior and employ various strategies, such as meta-motivational strategies (Pekrun, 2006), which may drive them to utilize AI tools to meet personal needs (personalization affordances) and interpret AI feedback as realistic and supportive to sustain motivation and engagement (social presence affordances). Conversely, negative emotions can narrow learners’ focus or even increase task-irrelevant thinking (Pekrun, 2006) and lead to their reliance on external regulation, which may decrease their engagement in using AI and overlook AI capabilities. Therefore, learners in the profile with high positive and low negative emotions are hypothesized to perceive the highest level of interactivity, personalization, convenience, and social presence affordances.

As for the antecedents (i.e., ERSs) of emotion profiles, the CVT proposes methods of emotion regulation, such as appraisal-oriented regulation. However, it does not explicitly differentiate ERSs. Therefore, the PMER (Gross, 2015) was integrated to specify the effects of two ERSs (cognitive reappraisal and expressive suppression) on emotion profiles. Cognitive reappraisal has demonstrated adaptive effects across sociocultural and technological contexts (Alqarni, 2024; S. Guo et al., 2025). Specifically, cognitive reappraisal occurs early in the emotion-generative process and enables learners to positively reframe their control over and value of learning activities before emotional responses are fully activated (Gross, 2015; Harley et al., 2019). Therefore, it can effectively increase positive emotions and reduce negative ones across Western and Eastern cultures, as well as F2F and IDLE settings (Alqarni, 2024; S. Guo et al., 2025). In contrast, expressive suppression presents a more culturally contingent pattern (Gross, 2015). In Western individualistic contexts where emotional authenticity is emphasized, suppression is generally associated with intensified negative emotions (Butler et al., 2007). However, research in Asian collectivist cultures, including China, has revealed a more nuanced picture. On the one hand, similar to the findings in Western research, expressive suppression depletes additional cognitive resources to conceal natural emotion displays and disconnects learners from authentic emotional experiences, which may be less effective in alleviating negative emotions, leaving negative emotions lingering at a high level (Jiang et al., 2025). On the other hand, due to cultural norms that emphasize interpersonal harmony and self-restraint, expressive suppression may also yield positive emotions by preventing conflict, protecting face, and sustaining smooth social interactions (Fernandes & Tone, 2021). This culturally grounded emotional benefit can be strengthened in IDLE settings with reduced social evaluation and active IDLE engagement (S. Guo et al., 2025), allowing cognitive resources to be allocated to cognitive reappraisal, thereby enhancing positive emotions. It is thus hypothesized that cognitive reappraisal strongly predicts profiles featuring high positive and low negative emotions, while expressive suppression largely predicts profiles featuring high negative and high positive emotions in the present study.

### 2.2. Emotions and Emotion Profiles

Emotions and their profiles were at the core of the present study. In recent years, emotion research in IDLE has garnered increasing scholarly attention (Lee et al., 2024; G. Liu et al., 2025a; Zou et al., 2025a). Defined as “self-directed, naturalistic, digital learning of English in unstructured, out-of-class environments, independent of a formal language program” (Lee, 2019, p. 116), IDLE affords learners with numerous opportunities to practice English anywhere and anytime, transcending the traditional classroom setting (Soyoof et al., 2023). IDLE is crucial because of the positive associations between engagement in such a context with EFL learners’ willingness to communicate (Lee et al., 2024), motivation (G. Liu et al., 2025c), critical thinking (Feng & Yang, 2025), flow (Fang et al., 2025), and English achievement, such as reading performance (Rezai et al., 2025) and oral proficiency (Guan et al., 2025). The emergence of AI, especially generative AI since late 2022, improves learners’ willingness to communicate (H. Liu & Fan, 2025), increases learning engagement (F. Guo et al., 2025), and maximizes the benefits of IDLE (G. Liu et al., 2025a).

Emotions in AI-mediated IDLE yield a high level of uniqueness due to the learning setting and medium. On the one hand, given the features of instant interactivity, personalized feedback, and self-directed learning without external stress, learners in this context can experience high positive emotions such as enjoyment (Lee et al., 2024; G. Liu et al., 2025c). On the other hand, the context yields a more complex impact on negative emotions. Take anxiety, the most-researched negative emotion, for example. Different from the classroom context with anxiety sources such as F2F communication, IDLE provides learners with a low affective filter environment owing to the lack of teacher and peer pressure, anonymity in learning, availability of abundant resources, and asynchronous communication (Lee, 2022). Recent research has generally revealed the negative association between IDLE engagement and anxiety (Lee et al., 2024). However, the technophobia aroused by AI has also become a salient source of EFL learners’ anxiety (Q. Wang et al., 2025).

To understand emotions in AI-mediated IDLE more comprehensively, the present study incorporates two other frequently experienced emotions (hope and disappointment). Hope arises when learners perceive attainable goals and possess confidence in their ability to achieve them (Pekrun, 2006). It is characterized by goal-directed pathways thinking (the generation of plausible routes to achieve goals) and agency thinking (the perceived ability to adopt and adapt one’s pathways to achieve goals even in face of impediments) (Snyder, 2002) and positively relates to self-regulated strategies (Teng & Pan, 2024), English learning effort (H. Liu et al., 2025b), and language performance (Shao et al., 2020). In AI-mediated IDLE, learners can adopt various tools for specific purposes, such as iFlytek Spark for oral practice (Guan et al., 2025) and ChatGPT chatbots for learning resources recommendation (G. Liu et al., 2025a). These tools enable learners to find out ways to solve problems, which can thus strengthen their pathways and agency thinking, making it possible to reinterpret their previous failing learning experiences in a more constructive light, and therefore, foster hope.

Disappointment refers to a feeling of dissatisfaction and psychological loss (Van Dijk & Zeelenberg, 2002), which is typically triggered by discrepancies between anticipated outcomes and actual experiences (Pekrun, 2006). It can exert negative effects on EFL learning, including demotivation (Tang & Hu, 2022) and unsatisfying learning outcomes (Mahfoodh, 2017). Compared with anxiety, disappointment is less investigated because its deactivating feature renders it less salient and more difficult to observe (Pekrun, 2006). However, it is a pervasive emotion frequently experienced by EFL learners (Mahfoodh, 2017), especially by beginners of EFL learning (Nakamura, 2018). The intensity and frequency of disappointment may be even more pronounced in the AI-mediated IDLE context. On the one hand, learners have high expectations from powerful AI tools that provide abundant resources, instant interaction, and personalized feedback (G. Liu et al., 2025a). Nevertheless, due to unsatisfying interface design (Zou et al., 2026), AI hallucination (G. Liu et al., 2025a), robotic feedback (Xin & Derakhshan, 2024), pronunciation recognition problems (Jeon, 2022), insufficient useful suggestions (Guan et al., 2025), and limited AI literacy (Yang & Zhao, 2024), learners may feel unsatisfied with AI-generated responses and fail to achieve the anticipated positive outcomes, which can trigger disappointment in AI-mediated IDLE.

Research on emotions in IDLE remains largely variable-centered, which assumes participant homogeneity and focuses on mean levels of emotions and their links to variables, such as willingness to communicate (Lee et al., 2024) and motivation (Zou et al., 2025a). Emotion profile research, adopting a person-centered approach, assumes subject heterogeneity and reveals the nuanced patterns in learner characteristics (Zhu et al., 2024). Existing emotion profiles among EFL learners have mainly been conducted in the in-class F2F learning context (Tsang & Yeung, 2024; Y. Wang & Xu, 2024; Zhu et al., 2024) and the pre-exam setting (Shi & Wang, 2025), which have generally revealed at least three emotion profiles, namely, the moderate type (moderate positive and moderate negative emotions), the positive type (high positive and low negative emotions) and the negative type (high negative and low positive emotions), with different proportions. A high positive and high negative emotions profile was less commonly found (Shi & Wang, 2025; Tsang & Yeung, 2024). Table 1 summarizes the emotion profile research with context, profile types, and key results. Due to the context-specific feature of emotions, whether their composition and distribution in the F2F setting can be generalized to technology-enhanced settings, such as AI-mediated IDLE, awaits scholarly attention. Examining emotion profiles in IDLE can thus clarify differences between F2F and IDLE contexts while complementing variable-centered approaches.

### 2.3. Emotion Profiles and Perceived AI Affordances

Based on the emotion profiles identified in AI-mediated IDLE, it is necessary to further examine their associations with outcome variables to discern the optimal profile. As shown in Table 1, prior studies have shown that profiles with high positive and low negative emotions are linked to stronger motivation in listening, speaking, reading, and writing (Tsang & Yeung, 2024; Y. Wang & Xu, 2024); writing buoyancy (Y. Wang & Xu, 2024); higher achievement in English sub-skills (Tsang & Yeung, 2024; Y. Wang & Xu, 2024); and overall English performance (Shi & Wang, 2025). However, further research is required to explore how emotion profiles relate to AI-related outcomes in IDLE, particularly AI affordances.

Originating from ecological psychology, the concept of “affordance” refers to the action possibilities that emerge through the interaction between an organism and its environment (Gibson, 1986). It has been adapted in human–computer interaction to denote the functional possibilities of AI tools (Xu & Li, 2024). Research has concurred that AI affordances are a multidimensional construct encompassing pedagogical affordances (enabling knowledge construction, scaffolding and feedback) (Jeon, 2022), personalization affordances (supporting tailored learning plans, goals and contents) (Xu & Li, 2024), and learning strategies affordances (facilitating effective learning and correct use of strategies) (R. Zhang et al., 2025). Considering that AI affordances must be perceived by the user to transform into actual functionality, perceived AI affordances, referring to the perceived possibilities that AI tools can provide in IDLE, were adopted in the present study.

Perceived AI affordances are a key outcome variable in IDLE, as empirical evidence has verified their close links with EFL learners’ satisfaction with AI tools and continuance intention to use AI tools (Huang & Zou, 2024), as well as cognitive and behavioral engagement (Huang et al., 2025; Xu & Li, 2024). Previous studies have mainly examined how perceived AI affordances influence emotions (Jeon, 2022; J. Zhang et al., 2024). The results showed that learners’ perceived AI affordances positively predicted flow (with enjoyment as one sub-dimension) (J. Zhang et al., 2024) and enjoyment in EFL learning (Cui et al., 2025) and exerted mixed effects on English speaking anxiety—reducing it by freeing learners from speaking in front of others while increasing it when speech misrecognition occurred (Jeon, 2022). Emerging evidence indicates that emotions are not mere by-products of AI interaction. Instead, they shape users’ perceptions of what AI tools can do for them (E. L. Cohen & Myrick, 2023). In the AI-mediated EFL learning context, existing research has examined how emotions shape perceived usefulness of AI tools (Dou et al., 2025; Huang & Zou, 2024; Tram et al., 2024; C. Wang et al., 2025), rather than perceived AI affordances. For example, Huang and Zou (2024) revealed that enjoyment in interacting with AI technology was positively associated with willingness to communicate, which in turn predicted the perceived usefulness of AI tools. Extending this line of inquiry into the AI-mediated IDLE context, C. Wang et al. (2025) revealed that learners’ perceived enjoyment in using multimodal AI tools positively predicted perceived usefulness of the technology. Considering that the perceived usefulness of AI tools “indicates users’ perceptions regarding technology affordances” (Huang & Zou, 2024, p. 2), it can be hypothesized that emotions can predict learners’ perceived AI affordances. A further inquiry into the effects of emotion profiles on perceived AI affordances helps detect the optimal emotional state for enhancing perceived AI affordances.

### 2.4. Emotion Regulation Strategies and Emotion Profiles

Considering the potential different roles of emotion profiles in influencing perceived AI affordances, it is necessary to promote students’ entry into an optimal emotion profile with the highest level of perceived AI affordances by investigating emotion profiles’ antecedents, one of which is ERSs. Cognitive reappraisal and expressive suppression are the two most widely compared strategies due to their substantial and contrasting impacts on emotional and learning outcomes (Gross & John, 2003). Cognitive reappraisal has been generally regarded as an adaptive strategy in EFL learning (Jiang et al., 2025), as it can increase positive emotions (Alqarni, 2024) while reducing negative ones (Gross & John, 2003). Specifically, cognitive reappraisers tend to have a positive attitude towards learning and positively perceive their English proficiency (Yüksel et al., 2025), enabling them to experience positive emotions.

In contrast to cognitive reappraisal, expressive suppression demonstrated mixed effects on emotions. Expressive suppression was shown to decrease positive emotions and heighten negative emotions, as simply reducing the outward display of emotions rarely eases inner negative feelings (Yüksel et al., 2025). Instead, it can create self-incongruence and deplete cognitive resources necessary for optimal performance (Gross & John, 2003). However, expressive suppression may not be completely unfavorable because it can positively relate to motivation (Namaziandost & Rezai, 2024) and behavioral engagement (Zare et al., 2025), which makes it possible for learners to increase positive emotions. The mixed impacts of expressive suppression on emotions can be attributed to cultural norms and contextual factors. Cultural norms critically shape whether expressive suppression undermines or sustains positive emotions. Different from Western samples (Gross & John, 2003), learners in collectivist cultures like Iran (Zare et al., 2025) and China (S. Guo et al., 2025) view expressive suppression as socially adaptive, helping maintain group harmony and learning engagement without necessarily diminishing positive emotions. In addition to cultural norms, one salient contextual factor influencing emotions is the learning environment. Specifically, in technology-mediated environments with tools such as supportive and adaptive AI learning systems (Namaziandost & Rezai, 2024), the negative impact of expressive suppression on emotions can be buffered, as alternative channels fulfill emotional needs.

The two strategies have been examined in the digital learning context (S. Guo et al., 2025; Yang & Zhao, 2024; Yüksel et al., 2025). For example, Yang and Zhao (2024) revealed that EFL learners regulated AI-induced emotions by focusing on the utility of AI tools (cognitive change) or by concealing feelings due to the perception that AI tools have no emotions (emotion suppression). In one IDLE-situated study, S. Guo et al. (2025) showed that learners with a high level of cognitive reappraisal tend to actively engage in IDLE activities, and the active IDLErs reported a higher level of expressive suppression compared with the passive IDLErs. Despite the importance of the two strategies in digital learning, how they predict emotion profiles in AI-mediated IDLE remains underexplored.

### 2.5. Research Questions

After the review of relevant theories and empirical studies, the following gaps have been identified. First, due to the uniqueness of emotions across sociocultural and technological milieus and the need to research the heterogeneity of emotions among EFL learners, a person-centered approach to examine emotion profiles in AI-mediated IDLE is warranted. Second, despite empirical evidence regarding the impact of emotions on perceived AI usefulness (Huang & Zou, 2024; C. Wang et al., 2025), the possible relationship between emotion profiles and perceived AI affordances awaits examination. Third, despite the predictive effects of cognitive reappraisal and expressive suppression on emotions (Alqarni, 2024; Gross & John, 2003), the impact of the two strategies on emotion profiles merits further investigation. To address these gaps, the present study draws upon the CVT and the PMER and aims to answer the following three research questions:

RQ1: What are Chinese EFL learners’ latent profiles concerning enjoyment, hope, anxiety, and disappointment in AI-mediated IDLE?

RQ2: Do Chinese EFL learners’ perceived AI affordances differ across their emotion profiles?

RQ3: What are the predictive effects of cognitive reappraisal and expressive suppression on Chinese EFL learners’ emotion profile memberships?

## 3. Methodology

### 3.1. Participants

Via convenience and purposive sampling, 613 EFL undergraduates from nine universities with AI experience in IDLE were recruited. Convenience sampling was adopted primarily for its practicality and efficiency in accessing participants, and purposive sampling was integrated to strategically select participants that covered key demographic dimensions (Dörnyei & Csizér, 2012). The combination of the two sampling methods thus balances easy accessibility and targeted representativeness, enabling the present study to recruit a large cohort of participants with AI experience in IDLE across diverse demographic backgrounds, as shown in Table 2. Specifically, the sample included 3 Double First-Class universities^1^ and 6 non-Double First-Class universities across North China, Northwest China, Central China, South China, and East China. The sample included 372 males and 241 females, aged 15–32 (*M* = 19.65, *SD* = 1.72). The numbers of participants from Year 1 to Year 4 were 272, 181, 107 and 53, respectively. There were 139 participants majoring in linguistics, 99 in humanities other than linguistics, 351 in science and engineering, and 24 in medicine. Participants self-rated their English proficiency at an intermediate level (*M* = 6.27, *SD* = 1.57, scale: 1–10). All the participants reported using both generative and non-generative AI tools (e.g., iWrite, DeepL, ChatGPT, Ernie Bot) for diverse purposes, including English writing, reading, oral communication, vocabulary, and grammar learning. Given the demographic diversity of the participants in institutional tiers, geographic regions, age, gender, grade, major, and self-rated English proficiency, the sample captured substantial heterogeneity among Chinese EFL learners engaged in AI-mediated IDLE.

### 3.2. Instruments

The questionnaire began with participants’ background information (e.g., gender, age, grade, major, self-rated English proficiency, AI tools used in IDLE and purposes), followed by 6 adapted scales to measure participants’ ERSs, emotions and perceived AI affordances in AI-mediated IDLE (see the Supplementary Materials for the scales). Contextualizing expressions such as “when using AI tools to learn English outside class” were emphasized. The original English instruments (ERSs, anxiety, hope and disappointment) were translated into Chinese and back-translated to ensure accuracy. The two authors, both specialized in applied linguistics, independently translated the original English items into Chinese and subsequently discussed the translations to reach a consensus on the final version. This Chinese version was then back-translated into English by an applied linguistics professor with extensive EFL teaching experience. Comparison was performed between the back-translated version and the original English instruments, with discrepancies examined item by item. Through iterative discussions, the Chinese version was refined until equivalence of meaning was achieved. Finally, the translated scales were piloted with 25 EFL learners, whose feedback regarding language clarity and content understanding was incorporated to ensure full comprehension and contextual appropriateness. Responses were rated on a five-point Likert scale from 1 (completely disagree) to 5 (completely agree).

#### 3.2.1. Emotion Regulation

Cognitive reappraisal and expressive suppression were measured via the emotion regulation questionnaire (Gross & John, 2003). It consists of 10 items, with 6 items assessing cognitive reappraisal (e.g., When I want to feel more positive emotion, I change the way I’m thinking about the situation) and 4 items measuring expressive suppression (e.g., I control my emotions by not expressing them). The emotion regulation questionnaire has been applied in the Chinese context with good reliability (S. Guo et al., 2025). In the present study, confirmatory factor analysis (CFA) results revealed the high structural validity of the questionnaire (*x*^2^/*df* = 2.19; CFI = 0.98; TLI = 0.98; SRMR = 0.03; RMSEA = 0.04). The Cronbach’s *α* values were 0.82 for cognitive reappraisal and 0.86 for expressive suppression, indicating good reliability.

#### 3.2.2. Achievement Emotions

Achievement emotions were measured via the scales of enjoyment (Li et al., 2018), hope (Pekrun et al., 2011), anxiety (Q. Wang et al., 2025), and disappointment (Pekrun et al., 2023). The composite questionnaire consists of five items for enjoyment (e.g., I’ve learnt interesting things), six items for hope (e.g., I have an optimistic view toward studying), three items for anxiety (e.g., I get a sinking feeling when I think of trying to use the large language models), and four items for disappointment (e.g., I feel disappointed that I did not succeed). The scales of enjoyment and anxiety were developed among Chinese EFL learners with satisfying reliability (Li et al., 2018; Q. Wang et al., 2025). The hope scale has been adopted in the Chinese context with high reliability and validity (Shao et al., 2020), and the disappointment scale has been applied among university students from various countries, including the United States, Canada, and Germany, showing acceptable reliability and validity (Pekrun et al., 2023). The model fit in the present study was satisfying (*x*^2^/*df* = 2.94; CFI = 0.96; TLI = 0.95; SRMR = 0.04; RMSEA = 0.06). The reliabilities of the four sub-scales of enjoyment, hope, anxiety, and disappointment were 0.79, 0.86, 0.86, and 0.91, respectively.

#### 3.2.3. Perceived AI Affordances

Perceived AI affordances were assessed by the AI affordances questionnaire validated by Xu and Li (2024) among Chinese EFL learners. It comprises 13 items across four dimensions, including interactivity affordance (e.g., AI tools can continuously respond to the instructions I give them), personalization affordance (e.g., AI tools can dynamically adjust the English learning content according to my personal situation), convenience affordance (e.g., I can learn English anytime, anywhere with the AI tools), and social presence (e.g., I feel encouraged and supported by the AI’s responses). In the present study, the model demonstrated good model fit (*x*^2^/*df* = 3.01; CFI = 0.96; TLI = 0.95; SRMR = 0.03; RMSEA = 0.06). Its internal consistency, measured by Cronbach’s alpha, reached 0.89.

### 3.3. Data Collection and Analysis

Questionnaires were distributed to EFL teachers who then helped administer the questionnaires to their 1296 students via an online survey platform (www.wjx.cn, accessed on 23 December 2024), and 714 students submitted their answers, with the response rate being 55%. The students were informed of the purpose and procedure of the study and were assured of the anonymity and that there would be no adverse impact on their academic record at school. Those who intended to participate signed consent forms. The researchers checked the students’ answers to identify careless responses (e.g., failing trap questions). Bonus money (2 yuan, or about 0.28 dollars for each participant) was then given to the eligible participants to acknowledge their participation. After excluding careless responses, 613 valid questionnaires were retained for analysis. Preliminary analyses assessed descriptive statistics, normality, reliability in SPSS 26, and validity in Mplus 8.3. The construct validity of the measurement model is considered acceptable when the indicators meet the criteria (*x*^2^/*df* < 3, CFI > 0.90, TLI > 0.90, SRMR < 0.07, RMSEA < 0.08) (Hair et al., 2014).

As for RQ1, latent profile analysis (LPA) was conducted via Mplus 8.3 to categorize the participants into different groups based on their raw scores of emotions. Bivariate residuals were inspected to assess the local independence assumption. A bivariate residual value lower than 4 indicates that the residual association between variables is adequately explained by the latent classes, supporting the assumption of local independence (Magidson et al., 2020). In the present study, all bivariate residual values were below this threshold, indicating that the local independence assumption was met. In model selection, the indices of AIC, BIC, aBIC, LMR, BLRT, entropy, and the percentage of the smallest profile were considered (Ferguson et al., 2019). Lower values of AIC, BIC and aBIC indicate a higher model fit (Ferguson et al., 2019). The *p*-values of LMR and BLRT are below 0.05, which indicates that the *k*-profile model outperforms the *k*-1 model (Nylund et al., 2007). An entropy value higher than 0.80 indicates highly discriminating profiles and relatively low classification uncertainty (Ferguson et al., 2019; Tein et al., 2013), providing supplementary support for model selection. Finally, each profile must represent at least 5% of the sample for theoretical interpretability (Nylund et al., 2007).

For RQ2, the BCH command in Mplus 8.3 was performed to examine the differences in students’ perceived AI affordances across their emotion profiles (Asparouhov & Muthén, 2021). Wald chi-square tests were executed to determine the statistical significance (S. Guo et al., 2025). In addition, the effect sizes of the differences were evaluated according to Cohen’s *d*, with values of 0.20, 0.50, and 0.80 defined as small, medium, and large effect sizes, respectively (J. Cohen, 1988).

For RQ3, to investigate the effects of ERSs on students’ profile membership, cognitive reappraisal and expressive suppression were added as predictors in the LPA model, respectively, using the R3STEP command in Mplus 8.3 (Asparouhov & Muthén, 2014). To reduce the potential confounding effects of demographic factors, gender, age, and self-rated English proficiency were included as covariates in the analyses, following the prior literature (S. Guo et al., 2025; Shao et al., 2020). Odds ratios (ORs) were generated to evaluate the likelihood of participants assigned to different profiles based on cognitive reappraisal and expressive suppression, with an OR higher than 1 indicating a greater likelihood of membership in the comparison group versus the reference group (Zhu et al., 2024).

## 4. Results

### 4.1. Preliminary Analyses

Descriptive analysis (see Table 3) indicates that the participants possessed a high level of positive emotions (enjoyment and hope), cognitive reappraisal, and perceived AI affordances. Negative emotions (anxiety and disappointment) and expressive suppression remained at a moderate level. The skewness and kurtosis values fell between −2 and 2, indicating a normal distribution (Kunnan, 1998).

### 4.2. Latent Profile Models of Emotions in AI-Mediated IDLE

#### 4.2.1. Identification of the Suitable Profile Model

The present study investigated the profile solutions from one to five based on the mean values of the four emotions. Table 4 shows the fit indices of the five models. Although AIC, BIC, and aBIC decreased with the inclusion of more profiles, improvements were marginal beyond three. For the three-profile model, it demonstrated a dramatic increase in fitness compared with the two-profile model (ΔBIC = −393.75), with the values of the LMR*p* and BLRT*p* tests being significant. In addition, with an entropy value of 0.81, it featured low classification uncertainty. Additionally, the three-profile solution displayed adequate participants for each profile. Finally, the three profiles reflected the coexistence of positive and negative emotions and their distinct intensities, which were consistent with the CVT.

Although the four-profile and five-profile solutions had higher values of entropy (0.83 and 0.82, respectively), they reported less trivial fit gains (four-profile solution: ΔBIC = −105.53; five-profile solution: ΔBIC = −83.63) compared with the three-profile solution. In addition, the values of LMR*p* were insignificant (four-profile model: 0.088 > 0.05; five-profile model: 0.241 > 0.05). Therefore, the three-profile model was selected due to model fitness superiority and theoretical interpretability.

#### 4.2.2. Description of the Selected Profile Model

**Figure 1:**
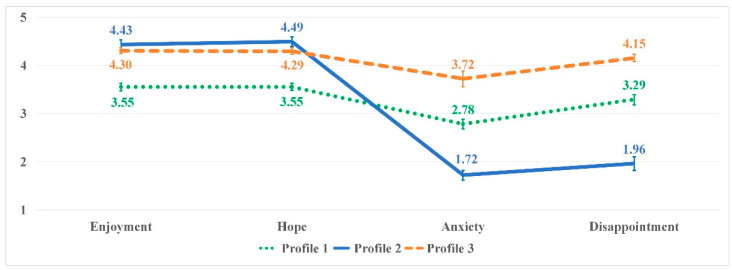
Mean scores of emotions for the three-profile solution with error bars. Note: Profile 1 = moderate positive and moderate negative emotions group; Profile 2 = high positive and low negative emotions group; Profile 3 = high positive and high negative emotions group.

The mean scores of the four emotions are presented in Figure 1. Profile 1 (N = 264, 43%) was the largest group with moderate enjoyment (*M* = 3.55, 95% CI: [3.47, 3.63]), hope (*M* = 3.55, 95% CI: [3.48, 3.63]), anxiety (*M* = 2.78, 95% CI: [2.68, 2.88]), and disappointment (*M* = 3.29, 95% CI: [3.18, 3.39]). Profile 2 (N = 128, 21%) was the smallest group, including participants who possessed high enjoyment (*M* = 4.43, 95% CI: [4.32, 4.53]) and hope (*M* = 4.49, 95% CI: [4.39, 4.59]), as well as low anxiety (*M* = 1.72, 95% CI: [1.61, 1.82]) and disappointment (*M* = 1.96, 95% CI: [1.82, 2.10]). Profile 3 (N = 221, 36%) comprised the second largest group, featuring high enjoyment (*M* = 4.30, 95% CI: [4.24, 4.37]), hope (*M* = 4.29, 95% CI: [4.23, 4.36]), anxiety (*M* = 3.72, 95% CI: [3.55, 3.88]), and disappointment (*M* = 4.15, 95% CI: [4.08, 4.23]).

Based on the distribution of positive and negative emotions, Profiles 1, 2, and 3 were named “moderate positive and moderate negative emotions group”, “high positive and low negative emotions group” and “high positive and high negative emotions group”, respectively. Despite differences in emotion distribution, all three profiles exhibited higher levels of positive emotions than negative emotions, suggesting the effectiveness of AI tools in fostering constructive emotional experiences in IDLE.

### 4.3. Differences in Perceived AI Affordances Across Emotion Profiles

BCH analysis showed the means and differences in perceived AI affordances across the three profiles (Table 5). Learners in Profile 2 reported the highest level of perceived interactivity affordance (*M* = 4.44), significantly higher than those in Profile 1 (*M* = 3.76, *p* < 0.001), with a large effect size, and Profile 3 (*M* = 4.21, *p* < 0.001), with a small effect size. Learners in Profile 3 perceived significantly higher levels of interactivity affordance than those in Profile 1 (*p* < 0.001), with a large effect size. Similarly, learners in Profile 2 reported the highest level of perceived convenience affordance (*M* = 4.45), significantly higher than those in Profile 1 (*M* = 3.72, *p* < 0.001), with a large effect size, and Profile 3 (*M* = 4.27, *p* = 0.002), with a small effect size. Learners in Profile 3 perceived significantly higher levels of convenience affordance than those in Profile 1 (*p* < 0.001), with a large effect size.

As for perceived personalization affordance, learners in Profile 2 (*M* = 4.35) and Profile 3 (*M* = 4.26) scored significantly higher than those in Profile 1 (*M* = 3.50, *p* < 0.001), with large effect sizes. In a similar vein, learners in Profile 2 (*M* = 4.24) and Profile 3 (*M* = 4.21) perceived significantly higher levels of social presence affordance than those in Profile 1 (*M* = 3.44, *p* < 0.001), with large effect sizes. Learners in Profile 2 reported higher levels of perceived personalization and social presence affordances than those in Profile 3, albeit not to a statistically significant extent. In other words, learners in Profile 2 scored the highest on all the dimensions of perceived AI affordances in IDLE, followed by those in Profile 3, and students in these two profiles significantly outperformed their counterparts in Profile 1.

### 4.4. The Effects of ERSs on Emotion Profiles

Multinomial logistic regression, controlling for gender, age and self-rated English proficiency, was performed to examine the predictive effects of cognitive reappraisal and expressive suppression on participants’ emotion profile memberships. As shown in Table 6, when using Profile 2 as the reference group, learners who possessed higher levels of cognitive reappraisal had a significantly lower probability of entering Profile 1 than Profile 2 (*p* < 0.001, OR = 0.03 [0.01, 0.09]), and a lower but not significant probability of membership in Profile 3 than Profile 2 (*p* = 0.146, OR = 0.62 [0.32, 1.23]). With Profile 3 as the reference group, learners high in cognitive reappraisal were less likely to enter Profile 1 than Profile 3 (*p* < 0.001, OR = 0.04 [0.01, 0.12]). Taken together, learners who had higher levels of cognitive reappraisal were the least likely to be grouped into Profile 1. Comparatively, when learners possessed higher cognitive reappraisal, they were more likely to enter Profile 2 and Profile 3. This suggested that cognitive reappraisal was a strong predictor of profiles featuring high positive emotions (Profiles 2 and 3), with the strongest effect on the high positive and low negative emotions profile (Profile 2).

Regarding the predictive effect of expressive suppression on profile membership, results revealed that learners with higher levels of expressive suppression were significantly more likely to enter Profile 1 (*p* = 0.018, OR = 1.71 [1.28, 2.28]) and Profile 3 (*p* < 0.001, OR = 3.02 [2.30, 3.95]) compared with Profile 2. When Profile 3 was treated as the reference group, learners with higher levels of expressive suppression were significantly less likely to enter Profile 1 than Profile 3 (*p* < 0.001, OR = 0.57 [0.43, 0.76]). Overall, expressive suppression was the strongest predictor of membership in the high positive and high negative emotions profile (Profile 3), followed by Profile 1, with the weakest in Profile 2.

## 5. Discussion

### 5.1. Emotion Profiles in AI-Mediated IDLE

As for RQ1, the LPA results identified three emotion profiles in AI-mediated IDLE. The three profiles (Profile 1 = moderate positive and moderate negative emotions group, 43%; Profile 2 = high positive and low negative emotions group, 21%; Profile 3 = high positive and high negative emotions group, 36%) are consistent with the emotion combinations assumed in the CVT (Pekrun, 2006). Specifically, the prevalence of Profile 1 in the present study can be interpreted as a manifestation of the generally moderate emotional intensity outlined by Pekrun et al. (2023). In addition, the negative association between positive and negative emotions was described in the CVT, as reflected in “positive emotions and the absence of intense negative emotions” (Pekrun, 2006, p. 327), supporting the existence of Profile 2. Additionally, in certain achievement situations where “opportunities for success as well as the threat of failure” coexist (Pekrun, 2006, p. 321), both positive and negative emotions occur, supporting the existence of Profile 3. The present study empirically validates the CVT (Pekrun, 2006) and extends its applicability into the technology-assisted English learning context. More importantly, it complements the CVT by revealing the specific proportion of these emotion profiles in AI-mediated IDLE among Chinese EFL learners.

Profile 1 accounted for the largest proportion. Nearly half of the learners experienced a balanced emotion pattern, where enjoyment and hope coexisted with manageable levels of anxiety and disappointment. The predominance of this profile in AI-mediated IDLE echoes previous emotion profile studies situated in the in-class English writing setting (Y. Wang & Xu, 2024) and the pre-exam setting (Shi & Wang, 2025) among Chinese EFL learners. Its stability across different contexts may be attributed to East Asian culture, which emphasizes a moderate way of thinking and balanced emotions rather than extreme ones (Miyamoto & Ryff, 2011).

Profile 2 constituted the smallest proportion in AI-mediated IDLE, which aligns with Y. Wang and Xu (2024) and Zhu et al. (2024), revealing that the smallest number of learners possessed high positive and moderate/low negative emotions in the F2F learning. The consistency across different learning contexts may suggest that this emotional pattern was deeply rooted in the inherent challenges of foreign language learning. Learning English is a complex and difficult cognitive process, which may easily arouse negative emotions as learners are subject to no progress, fear of failure, and negative evaluation (Li et al., 2020). Negative emotions may be intensified when learning is exam-oriented, and success is highly valued in contexts like China (Zhu et al., 2024). Even when engaging in IDLE, learners’ purpose of performing well on high-stakes exams can also arouse performance pressure and strengthen negative emotions. The low prevalence of Profile 2 may also be attributed to the high self-regulatory demands inherent in the autonomous nature of AI-mediated IDLE (G. Liu et al., 2025a). Sustaining a state of high positive emotions and low negative emotions requires advanced self-regulated skills to consistently manage learning goals, monitor progress, and adapt strategies in an unstructured learning environment (Teng & Pan, 2024), which may pose a great challenge for most Chinese students who are accustomed to a teacher-directed culture.

Interestingly, the low positive and high negative emotions profile identified in previous emotion profile studies (Shi & Wang, 2025; Y. Wang & Xu, 2024; Zhu et al., 2024) was absent in the present study. Instead, our study revealed a rarely discussed emotion profile (Profile 3 with high positive and high negative emotions), comprising the second largest group. This was counterintuitive, as results generally revealed a negative correlation between positive and negative emotions (Pekrun et al., 2023; Shao et al., 2020). However, the result corroborated Shi and Wang (2025) and Tsang and Yeung (2024), showing that students experienced high enjoyment and high anxiety simultaneously, further indicating that positive and negative emotions were not in a seesaw relationship. The identification of this specific emotion profile may also reflect the complexity of AI-mediated IDLE, accentuating the context-specificity of emotion profiles. AI-mediated IDLE provides learners with instant interaction, personalized feedback, and autonomous and self-directed learning opportunities, without external pressure from teachers and peers, all of which may enhance their intrinsic motivation to learn (G. Liu et al., 2025c), thus improving their enjoyment and hope. At the same time, AI-mediated IDLE, due to the novelty and complexity of AI tools, can impose additional psychological burdens on learners, especially those with low AI literacy (Yang & Zhao, 2024; Q. Zhang et al., 2025) and introduce new stressors, such as unfamiliarity with AI tools, poor ability in effective use of AI tools, robotic feedback, repetitive contents, and unsatisfying answers (Jeon, 2022; Xin & Derakhshan, 2024; Yang & Zhao, 2024), all of which may exacerbate their negative emotions such as anxiety and disappointment.

The smallest proportion of Profile 2 (high positive and low negative emotions group), together with the relatively large proportion of Profile 3 (high positive and high negative emotions group), indicates that many Chinese learners in AI-mediated IDLE are not in an optimal emotional state. Although IDLE emphasizes learner autonomy (Lee, 2019), teachers continue to play a critical role in making pedagogical decisions that guide learners towards more emotionally adaptive states. Instructional designs can be strategically adjusted to enhance learners’ positive emotions while alleviating negative ones (Nakamura et al., 2024), particularly through process-oriented task design, appropriate task scaffolding, and explicit support for self-regulated learning and AI literacy. In this way, EFL learners may become more emotionally empowered even when engaging in AI-mediated IDLE.

Considering the sample characteristics, English learning and education policies, and technology adoption in China, the distribution and proportion of emotion profiles revealed in the present study should be interpreted as contextually situated. This context-specificity constitutes a potential limitation of the study, as it constrains the generalizability of the observed emotion profiles beyond the Chinese IDLE context. Although similar emotional patterns may emerge in settings that share comparable sociocultural values, policy orientations, and levels of technological integration, caution is warranted when extending the findings to contexts with divergent cultural norms (Miyamoto & Ryff, 2011), digital competence (He & Li, 2019), and pedagogical traditions (McInerney & King, 2017). Future studies are, therefore, called upon to conduct cross-context comparisons to examine the stability and variability of emotion profiles in different cultural, social and technological settings.

### 5.2. Perceived AI Affordances Across Different Emotion Profiles

Regarding RQ2, the present study revealed that students in Profile 2 (high positive and low negative emotions group) scored the highest on perceived AI affordances, followed by those in Profile 3 (high positive and high negative emotions group) and Profile 1 (moderate positive and moderate negative emotions group). This study highlights the need to distinguish emotion profiles and identifies the optimal one for targeted intervention. It also extends emotion profile research by validating a new outcome variable (i.e., perceived AI affordances) beyond motivation, writing buoyancy, as well as English sub-skill and overall achievement (Shi & Wang, 2025; Tsang & Yeung, 2024; Y. Wang & Xu, 2024; Zhu et al., 2024).

Given the conducive role of perceived AI affordances in the AI-mediated learning environment (Xu & Li, 2024), it can be summarized that Profiles 2, 3 and 1 were the optimal, the suboptimal and the least ideal, respectively. The optimal profile identified in the study lends empirical support to the CVT, proposing that high positive and low negative emotions enable learners to concentrate on the learning task, promote self-regulated learning and enhance learning engagement (Pekrun, 2006), which can provide learners with opportunities to explore the functions of AI tools and possibly perceive high levels of AI affordances. The high positive and low negative emotions yielded accumulated benefits, leading to the highest level of perceived AI affordances. The result also aligns with previous studies showing the facilitative effect of high positive and low negative emotions on IDLE engagement (Lee et al., 2024; G. Liu et al., 2024). As Y. Wang and Xu (2024) indicated, learners in the positive profile were more resilient and motivated than those in the moderate profile, which may further make it possible for them to perceive more AI affordances. Therefore, it is crucial for instructors to support learners in attaining the optimal Profile 2. Practically, this can be achieved by addressing negative emotions-arousing situations through teacher support, such as appropriate instructional design and effective ERSs instruction, thereby fostering emotional conditions that are most conducive to enhancing IDLE engagement.

The coexistence of high positive and negative emotions also guaranteed a relatively high level of perceived AI affordances, supporting the CVT, which assumes that negative emotions may not always exert negative effects (Pekrun, 2006). This is especially true for activating negative emotions (e.g., anxiety). Although they impair intrinsic motivation, extrinsic motivation can be induced to avoid potential failures (Pekrun et al., 2023). Therefore, learners with high activating negative emotions may still engage in the learning process and discover AI affordances to ensure success. The higher perceived AI affordances among learners in Profile 3 than Profile 1 can also be attributed to the role of arousal and attention narrowing in emotion. Emotions influence individuals’ appraisals of the urgency or importance of a task through arousal, with the high arousal amplifying an individual’s perception, evaluation and cognitive processing of the environment (Storbeck & Clore, 2008). Arousal occurs selectively rather than globally due to attention narrowing, as attention can be directed to high-priority and relevant cues rather than the low-priority and irrelevant ones (Mather & Sutherland, 2011). Therefore, when students experience a coexistence of high positive and high negative emotions, they may enter a state of high physiological and cognitive arousal, rendering them more attentive to high-priority and relevant cues such as interactivity and personalization in the AI-mediated learning environment, and therefore, perceiving more AI affordances.

Since the present study was conducted in the Chinese EFL context, where AI-mediated IDLE practices are increasingly normalized (G. Liu et al., 2025a), learners are more likely to perceive AI affordances with accumulated learning experience. High positive emotions, whether accompanied by low or high negative emotions, may function as a catalyst that mobilizes cognitive resources, sustains task engagement, and facilitates deeper interaction with AI tools, thereby enhancing learners’ perceptions of AI affordances across multiple dimensions. In other words, the strength of the observed associations may be contingent on the relatively high level of technological accessibility and AI adoption in the Chinese EFL setting. Therefore, despite the universal functional mechanism of emotions on cognitive and self-regulatory processes (Pekrun, 2006), the extent to which emotional profiles translate into affordance perceptions may be better generalized to countries and regions with similar technological readiness, as technological availability and exposure have been shown to fundamentally influence learners’ perceptions and behavioral tendencies towards AI (G. Liu et al., 2025b; Ma et al., 2024). Accordingly, future studies are needed to compare learners’ perceived AI affordances in IDLE across varying technological conditions and further examine the magnitude of the association between emotion profiles and perceived AI affordances.

### 5.3. The Predictive Effects of ERSs on Emotion Profiles

Concerning RQ3, results revealed that cognitive reappraisal predicted memberships in Profiles 2, 3, and 1 in a descending order in AI-mediated IDLE. The result supported the PMER, showing the positive role of cognitive reappraisal in enhancing positive emotions and decreasing negative emotions (Gross & John, 2003). In addition, our study adds nuance to the PMER by revealing that cognitive reappraisal did not uniformly bring about purely positive outcomes but may also lead to high levels of both positive and negative emotions. The strongest effect of cognitive reappraisal on Profile 2 aligns with prior research showing that this strategy effectively increased positive emotions and mitigated negative ones (Jiang et al., 2025). This further demonstrates the adaptive role of cognitive reappraisal (Gross & John, 2003). Learners who use cognitive reappraisal tend to enhance cognitive and behavioral engagement (S. Guo et al., 2025), which may increase their perceived control and value of the learning activity, making it easier for them to experience positive emotions. This can be especially obvious in AI-mediated IDLE, as AI tools often provide EFL learners with instant interaction, personalized feedback and a large database of authentic English learning materials (G. Liu et al., 2025a). This reinforces EFL learners’ sense of progress and competence, assisting them in reinterpreting their learning as controllable and valuable, therefore leading to high positive and low negative emotions.

The effect of cognitive reappraisal on high positive and high negative emotions was less anticipated. One possible explanation is related to the negative emotion intensity (Gross, 2015). The functioning of reappraisal calls for cognitive control (Buhle et al., 2014). When learners feel a high intensity of negative emotions, their cognitive resources become largely consumed by the emotion (Pekrun et al., 2023). Specifically, their cognitive repertoire for the learning tasks tends to be narrowed, making it hard for them to engage in cognitive reappraisal to reinterpret the learning experience. In addition, in a state of high emotional intensity, learners are more likely to adopt strategies such as distraction, which require relatively fewer cognitive resources (Sheppes et al., 2011). Due to the possible consumption of cognitive resources and shift to strategies such as distraction, cognitive reappraisal may become less effective, leaving the high negative emotion intensity lingering on (Gross, 2015).

In contrast to cognitive reappraisal, learners using expressive suppression were more likely to be categorized into Profile 3, followed by Profile 1 and then Profile 2 in AI-mediated IDLE, complementing the PMER by showing the possible adaptive feature of expressive suppression (Gross, 2015). The finding confirms previous studies that demonstrated that expressive suppression heightened negative emotions, as it did not resolve underlying feelings, which then continued to linger and accumulate (Gross & John, 2003). Also, the results indicated that learners employing expressive suppression frequently reported a high level of positive emotions, which contradicts previous findings revealing that expressive suppression decreased positive emotions (Gross & John, 2003; Kashdan & Breen, 2008). This inconsistency highlights a potential contextual boundary of the present finding. One possible explanation was the setting of the present study. Different from the traditional classroom setting, where emotion suppression is common due to social norms, particularly in the Asian culture, which emphasizes control over emotional expression (S. Guo et al., 2025), AI-mediated IDLE features the absence of immediate social judgment, which may change the mechanism of expressive suppression. In such a context, learners may not need to experience the cognitive cost of worrying about displaying positive emotions in front of others, which enables them to focus on learning tasks and enhance behavioral engagement (Zare et al., 2025). This heightened task engagement, in turn, increases learners’ perceived control over the learning activities and creates more opportunities for them to experience task success, which are critical antecedents of positive emotions. In addition, when expressive suppression is coupled with supportive AI-learning environments, its negative emotional impact may be buffered (Namaziandost & Rezai, 2024). Specifically, despite suppressing emotions, learners may not necessarily experience a decline in positive emotions, as their emotional needs can be met through other channels, such as the personalized and adaptive feedback they get from AI platforms. Taken together, the adaptive function of expressive suppression observed in this study may be shaped by the specific cultural and technological setting of AI-mediated IDLE. Therefore, it should be noted that generalizing the positive effect of expressive suppression on the suboptimal emotion profile (i.e., the high positive and high negative emotions profile) warrants caution, particularly due to cross-cultural differences with collectivism generally valuing emotion suppression while individualism preferring outward expression (Murata et al., 2013), as well as technological influence (S. Guo et al., 2025). Accordingly, it is suggested that future research could adopt comparative designs to systematically investigate the potential differences in the strength of the relationship between ERSs and emotion profiles across diverse cultural and technological contexts.

From a practical perspective, considering that learners who use cognitive reappraisal were the most likely to be categorized into the optimal emotional state, whereas those relying on expressive suppression were the least likely to attain this state, instructors should explicitly delineate the functions of the two ERSs. In particular, pedagogical support should prioritize helping learners reframe learning difficulties or unsatisfying AI interaction as opportunities, thereby fostering and sustaining high positive emotions. At the same time, given that cognitive reappraisal may become less effective under conditions of high negative emotion intensity (Gross, 2015), instructors can encourage emotion polyregulation (Ford et al., 2019). Learners may first adopt low-effort strategies, such as distraction, to reduce emotional intensity, and subsequently apply cognitive reappraisal once sufficient cognitive resources are restored, thus enhancing the effectiveness of cognitive reappraisal. For learners who tend to rely on expressive suppression, instructors are encouraged to examine whether this strategy enables them to experience positive emotions. When expressive suppression proves maladaptive, learners should be guided to supplement or replace it with more adaptive ERSs.

## 6. Conclusions, Implications and Limitations

Drawing upon the CVT and PMER, the present study was among the first attempts to investigate emotion profiles in AI-mediated IDLE and their associations with ERSs and perceived AI affordances. LPA results revealed three emotion profiles, including Profile 1 (moderate positive and moderate negative emotions group, the largest proportion), Profile 2 (high positive and low negative emotions group, the smallest proportion), and Profile 3 (high positive and high negative emotions group, the second largest proportion). BCH results revealed that learners in Profile 2 perceived the highest level of AI affordances, followed by Profile 3 and then Profile 1. Multinomial logistic regression analysis results demonstrated that cognitive reappraisal was the strongest predictor of membership in Profile 2, and expression suppression was the most linked with Profile 3.

The study yields a number of implications. Theoretically, it advances the literature by proposing a person-centered and context-specific integrative framework that combines the CVT and the PMER to unravel the emotion profiles, their critical antecedents (i.e., ERSs), and subsequent outcomes (i.e., perceived AI affordances) in the Chinese AI-mediated IDLE setting, facilitating an understanding of EFL learners’ emotional processes. Building upon the integrative framework, this study contributes to verifying and complementing the CVT and the PMER in four specific ways. First, although the CVT conceptually posits the coexistence of achievement emotions, the present study operationalizes this assumption through a person-centered approach, empirically quantifying the distribution and proportion of distinct emotion profiles in a specific cultural and technological setting. Particularly, the emotion profiles can be shaped by contextual factors, including the balanced way of thinking in Chinese culture, the exam-oriented and performance-driven learning culture in China, and the technology-enhanced learning environment. This suggests that CVT may require contextual calibration when applied to investigate emotions across contexts. Second, as for the impact of emotions, this study verifies the CVT’s proposition regarding the universal functional mechanism of emotions on cognitive and self-regulatory processes, specifically in the IDLE context in China. By validating the highest perceived AI affordances for Profile 2, it verifies the facilitative roles of positive emotions and constraining outcomes of negative emotions specified in the CVT. Third, as for the effects of ERSs on emotion profiles, it shows that cognitive reappraisal emerged as the strongest predictor of the optimal emotion profile, lending empirical support to the PMER, which posits the adaptive feature of cognitive reappraisal. However, the role of expressive suppression diverged from its generally negative characterization in Western frameworks. The present study particularly showed the positive effects of expressive suppression on positive emotions, suggesting that suppression may carry context-dependent benefits in Chinese collectivist culture and a less socially contingent AI-mediated IDLE setting. This complements the PMER by demonstrating that the adaptiveness of suppression is moderated by both cultural norms and technological environments rather than being universally maladaptive. Fourth, this study revealed that both ERSs were significant predictors to distinguish different emotion profiles, complementing the PMER in that the relationship between ERSs and emotion profiles may not remain linear and uniform. Overall, the present study contributes theoretically by proposing an integrated framework and situating it in a specific cultural and technological context to explain how emotions are regulated, configured, and function, thereby highlighting the need to critically examine the contextual boundaries and applicability of existing theoretical assumptions.

Pedagogically, considering the smallest percentage of the optimal emotion profile, teachers should understand learners’ emotional experiences associated with AI use in IDLE and their antecedents. Specifically, teachers can collect and analyze learner–AI collaboration logs and engage in follow-up interactions to identify students’ emotions and emotion-eliciting situations during the AI-mediated IDLE learning process. Teachers can also design specific AI agents and provide instructions on prompts to assist students in writing reflective journals, recognizing their emotional patterns in IDLE. Subsequently, targeted ERSs interventions can be conducted to enhance students’ positive emotions and mitigate negative ones. For example, teachers can foster cognitive reappraisal by integrating interactive tutorials and scenario-based simulations for out-of-class IDLE practice, while mitigating expressive suppression through facilitated group discussions and expressive writing tasks.

This study has the following limitations. First, due to the limited research scope, this study only investigated the profiles of four frequently experienced emotions. Future studies could incorporate more emotions to comprehensively understand emotion profiles in AI-mediated IDLE. Second, apart from the self-report questionnaires, which generally reflect trait emotions, empirical studies could also use measures such as the Electroencephalogram and AI emotion recognition to evaluate state emotions, which are more objective and instant. In addition, although the present study considered demographic factors (gender, age, and self-rated English proficiency) as control variables, future studies can incorporate other possible confounding variables, such as AI type (use of generative or non-generative AI tools) and AI engagement level, to further delineate the mediating roles of emotion profiles between ERSs and perceived AI affordances. Additionally, the study adopted a cross-sectional perspective and relied solely on quantitative self-report measures. Future research can adopt a longitudinal or mixed-methods design to examine and explain the dynamic evolutions of emotions and their associations with ERSs and perceived AI affordances. Finally, given the rapid evolution of AI technology, its uneven development across countries, pronounced global disparities in AI literacy, the distinctiveness of East Asian culture, and variations in participants’ academic backgrounds, the generalizability of the emotion profile patterns in IDLE and their associations should be treated with caution. Future replication studies are needed with emerging AI technologies and in different cultural and educational contexts.

## Figures and Tables

**Table 1:** A summary of the emotion profile research reviewed in the present study.

Study	Context	Emotion Profile Types	Key Results
Y. Wang and Xu (2024)	In-class EFL writing	Negative profile (P1, 43.0%) Positive profile (P2, 11.8%) Moderate profile (P3, 45.2%)	Students in P2 possessed the highest level of writing buoyancy, motivation and proficiency, followed by those in P3 and P1.
Zhu et al. (2024)	In-class EFL writing	Moderate-enjoyment/moderate-anxiety profile (P1, 24.3%) Moderate-enjoyment/low-anxiety profile (P2, 48.6%) High-enjoyment/moderate-anxiety profile (P3, 8.8%) Low-enjoyment/high-anxiety profile (P4, 18.3%)	An increase in imaginative capacity predicted students’ membership in P3 to a greater extent than in P4. Students in P2 scored the highest writing score, while those in P4 showed the worst writing performance.
Tsang and Yeung (2024)	In-class EFL learning	Negative emotion profile (P1, 21.43%) High enjoyment profile (P2, 46.93%) High enjoyment and anxiety profile (P3, 31.63%)	Students in P2 and P3 showed higher scores in various aspects of English proficiency and motivation than those in P1.
Shi and Wang (2025)	Pre-exam EFL learning	Positive-emotion-driven profile (P1, 31%) Bimodal-emotion-driven profile (P2, 56%) Negative-emotion-driven profile (P3, 13%)	Students in P1 and P2 achieved higher English scores than those in P3.

**Table 2:** Demographic information of participants (N = 613).

Demographic Information	Description	Number of Participants (Percentage)
Institutional tier	Double First-Class university	366 (60%)
	Non-Double First-Class university	247 (40%)
Geographic region	North China	286 (47%)
	Northwest China	50 (8%)
	Central China	118 (19%)
	South China	68 (11%)
	East China	91 (15%)
Gender	Male	372 (61%)
	Female	241 (39%)
Grade	Year 1	272 (44%)
	Year 2	181 (30%)
	Year 3	107 (17%)
	Year 4	53 (9%)
Major	Linguistics	139 (23%)
	Humanities other than linguistics	99 (16%)
	Science and engineering	351 (57%)
	Medicine	24 (4%)
Self-rated English proficiency	1–5	164 (27%)
	6–10	449 (73%)

**Table 3:** Descriptive statistics of the variables.

Variables	Mean	*SD*	Min	Max	Skewness	Kurtosis
Enjoyment	4.00	0.58	2.00	5.00	−0.45	0.40
Hope	4.02	0.58	2.00	5.00	−0.42	0.42
Anxiety	2.89	1.06	1.00	5.00	0.00	−0.99
Disappointment	3.32	1.00	1.00	5.00	−0.49	−0.61
Cognitive reappraisal	3.98	0.54	1.50	5.00	−0.52	1.23
Expressive suppression	3.25	0.98	1.00	5.00	−0.42	−0.56
Perceived AI affordances	4.00	0.53	1.77	5.00	−0.28	0.39

**Table 4:** Fit indices of models with different numbers of profiles.

Profile	AIC	BIC	ΔBIC	aBIC	LMR*p*	BLRT*p*	Entropy	Smallest Profile Percentage
1	5704.02	5739.37	-	5713.97	-	-	-	-
2	5336.94	5394.38	−344.99	5353.11	<0.001	<0.001	0.84	152 (25%)
**3**	**4921.10**	**5000.63**	**−393.75**	**4943.48**	**<0.001**	**<0.001**	**0.81**	**128 (21%)**
4	4793.48	4895.10	−105.53	4822.08	0.088	<0.001	0.83	45 (7%)
5	4687.76	4811.47	−83.63	4722.58	0.241	<0.001	0.82	58 (9%)

Note: The model that best fits the data is in bold.

**Table 5:** Means and differences in perceived AI affordances.

Outcome	Profile 1 Mean (*SE*)	Profile 2 Mean (*SE*)	Profile 3 Mean (*SE*)	Wald Chi-Square Test Results														
				Profile 1 vs. 2					Profile 1 vs. 3					Profile 2 vs. 3				
				*x* ^2^	*df*	*p*	95% CI	*d*	*x* ^2^	*df*	*p*	95% CI	*d*	*x* ^2^	*df*	*p*	95% CI	*d*
Interactivity	3.76 (0.04)	4.44 (0.05)	4.21 (0.04)	112.20	1	<0.001	[−0.81, −0.55]	−1.09	57.73	1	<0.001	[−0.57, −0.34]	−0.83	14.94	1	<0.001	[0.11, 0.34]	0.42
Personalization	3.50 (0.05)	4.35 (0.06)	4.26 (0.04)	109.70	1	<0.001	[−1.01, −0.69]	−1.31	138.64	1	<0.001	[−0.88, −0.63]	−1.37	1.48	1	0.223	[−0.06, 0.24]	0.13
Convenience	3.72 (0.04)	4.45 (0.05)	4.27 (0.04)	140.22	1	<0.001	[−0.85, −0.61]	−1.24	100.86	1	<0.001	[−0.66, −0.44]	−1.09	9.56	1	0.002	[0.07, 0.29]	0.33
Social presence	3.44 (0.05)	4.24 (0.07)	4.21 (0.04)	84.41	1	<0.001	[−0.98, −0.64]	−1.15	141.35	1	<0.001	[−0.90, −0.65]	−1.35	0.17	1	0.677	[−0.13, 0.20]	0.05

Note: Profile 1 = moderate positive and moderate negative emotions group; Profile 2 = high positive and low negative emotions group; Profile 3 = high positive and high negative emotions group. *d* = Cohen’s *d*.

**Table 6:** Profile membership predicted by cognitive reappraisal and expressive suppression.

Predictors	Profile 1 vs. Profile 2 (Profile 2: Reference Group)				Profile 3 vs. Profile 2 (Profile 2: Reference Group)				Profile 1 vs. Profile 3 Profile 3 (Reference Group)			
	*Est.*	*SE*	*p*	OR [95% CI]	*Est.*	*SE*	*p*	OR [95% CI]	*Est.*	*SE*	*p*	OR [95% CI]
Cognitive reappraisal	−3.70	0.77	<0.001	0.03 [0.01, 0.09]	−0.47	0.42	0.146	0.62 [0.32, 1.23]	−3.23	0.65	<0.001	0.04 [0.01, 0.12]
Expressive suppression	0.54	0.18	0.018	1.71 [1.28, 2.28]	1.10	0.16	<0.001	3.02 [2.30, 3.95]	−0.57	0.18	<0.001	0.57 [0.43, 0.76]
Gender	0.57	0.35	0.217	1.76 [0.99, 3.13]	0.26	0.33	0.488	1.29 [0.76, 2.21]	0.31	0.29	0.362	1.36 [0.84, 2.20]
Age	−0.04	0.10	0.647	0.96 [0.82, 1.12]	0.15	0.09	0.106	1.17 [1.01, 1.35]	−0.20	0.08	0.006	0.82 [0.72, 0.94]
Proficiency	−0.24	0.13	0.035	0.79 [0.64, 0.97]	−0.02	0.12	0.860	0.98 [0.81, 1.18]	−0.22	0.11	0.020	0.80 [0.68, 0.96]

Note: Profile 1 = moderate positive and moderate negative emotions group; Profile 2 = high positive and low negative emotions group; Profile 3 = high positive and high negative emotions group.

## Data Availability

The data presented in this study are available upon request from the corresponding author due to restrictions related to participant privacy concerns.

## Note

1 Double First-Class universities refer to the universities that are recognized as world-class universities or universities that possess first-class disciplines in China.

## Supplementary Materials

The following supporting information can be downloaded at https://www.mdpi.com/article/10.3390/bs16020283/s1. Achievement Emotions Questionnaire.
