# Supplementary material for: A Latent Profile Analysis of Emotions in AI-Mediated IDLE: Associations with Emotion Regulation Strategies and Perceived AI Affordances
Source: Behav Sci (Basel). 2026 Feb 15;16(2):283. doi: 10.3390/bs16020283 (PMC12938404; doi:10.3390/bs16020283)
Supplement: Supplementary file 1 [file behavsci-16-00283-s001.zip › behavsci-4067391-supplementary.pdf]

Supplementary Materials

A Latent Profile Analysis of Emotions in AI-Mediated IDLE: Associations with Emotion Regulation Strategies and Perceived AI Affordances

Achievement Emotions Questionnaire

Instruction

The items below are the emotions that you may experience when using AI tools to learn English outside class. Please recall your English learning situations with AI tools outside class and then indicate your level of agreement.

Enjoyment

1. I don’t get bored.
2. I enjoy it.
3. I’ve learnt interesting things.
4. I feel proud of my accomplishments.
5. It’s fun.

Hope

6. I have an optimistic view toward studying.
7. I feel confident when studying.
8. I feel confident that I will be able to master the material.
9. I feel optimistic that I will make good progress at studying.
10. The thought of achieving my learning objectives inspires me.
11. My sense of confidence motivates me.

Anxiety

12. I get a sinking feeling when I think of trying to use the large language models.
13. I hesitate to use the large language models for fear of making mistakes I cannot correct.
14. I have avoided the large language models because it is unfamiliar to me.

Disappointment

15. I am disappointed about my lack of accomplishments.
16. I feel disappointed that I did not succeed.
17. I am disappointed that I did not perform well.
18. I feel disappointed about my lack of ability.

Emotion Regulation Questionnaire

Instruction

The items below refer to the emotion regulation strategies you may use to regulate emotions aroused by using AI tools to learn English outside class. Please indicate your level of agreement.

Cognitive reappraisal

1. I control my emotions by changing the way I think about the situation I'm in.
2. When I want to feel less negative emotion, I change the way I'm thinking about the situation.
3. When I want to feel more positive emotion, I change the way I'm thinking about the situation.
4. When I want to feel more positive emotion (such as joy or amusement), I change what I'm thinking about.
5. When I want to feel less negative emotion (such as sadness or anger), I change what I'm thinking about.
6. When I'm faced with a stressful situation, I make myself think about it in a way that helps me stay calm.

Expressive suppression

7. I control my emotions by not expressing them.
8. When I am feeling negative emotions, I make sure not to express them.
9. I keep my emotions to myself.
10. When I am feeling positive emotions, I am careful not to express them.

Perceived AI affordances Questionnaire

Instruction

The items below concern your perceived AI usefulness when using AI tools to learn English outside class. Please indicate your level of agreement.

Interactivity affordance

1. AI tools can continuously respond to the instructions I give them.
2. AI tools can respond to my commands in a short period of time.
3. The interface of the AI tools is easy to use.

Personalization affordance

4. AI tools can dynamically adjust the English learning content according to my personal situation.

5. AI tools can provide me with customized learning plans and resources based on my personal situation.

6. AI tools can help me improve or achieve my goals based on my behavior or needs.

Convenience affordance

7. I can learn English anytime, anywhere with the AI tools.

8. AI tools are easy to use without complicated training or long adaptation process.

9. AI tools can automatically perform certain tasks, which improves my learning efficiency.

10. AI tools integrate multiple related services or functions, reducing the need to switch between different platforms.

Social presence

11. Interacting with the AI is like communicating with a real person.

12. I feel encouraged and supported by the AI's responses.

13. I am able to express my personality, opinions and feelings in an AI-empowered learning environment.
